# Hybrid System for *Ex Vivo* Hemorheological and Hemodynamic Analysis: A Feasibility Study

**DOI:** 10.1038/srep11064

**Published:** 2015-06-19

**Authors:** Eunseop Yeom, Yang Jun Kang, Sang Joon Lee

**Affiliations:** 1 Department of Mechanical Engineering, Pohang University of Science and Technology, Pohang, South Korea; 2 Department of Mechanical Engineering, Chosun University, Gwangju, South Korea

## Abstract

Precise measurement of biophysical properties is important to understand the relation between these properties and the outbreak of cardiovascular diseases (CVDs). However, a systematic measurement for these biophysical parameters under *in vivo* conditions is nearly impossible because of complex vessel shape and limited practicality. *In vitro* measurements can provide more biophysical information, but *in vitro* exposure changes hemorheological properties. In this study, a hybrid system composed of an ultrasound system and microfluidic device is proposed for monitoring hemorheological and hemodynamic properties under more reasonable experimental conditions. Biophysical properties including RBC aggregation, viscosity, velocity, and pressure of blood flows are simultaneously measured under various conditions to demonstrate the feasibility and performance of this measurement system. The proposed technique is applied to a rat extracorporeal loop which connects the aorta and jugular vein directly. As a result, the proposed system is found to measure biophysical parameters reasonably without blood collection from the rat and provided more detailed information. This hybrid system, combining ultrasound imaging and microfluidic techniques to *ex vivo* animal models, would be useful for monitoring the variations of biophysical properties induced by chemical agents. It can be used to understand the relation between biophysical parameters and CVDs.

Cardiovascular diseases (CVDs) may result from rheological^1^,^2^,^3^,^4^ and hemodynamic^5^,^6^ abnormalities. Among them, blood viscosity is considered as a crucial factor in determining the physiological and pathological conditions of CVDs^7^, because it augments flow resistance and wall shear stress, which stimulates the endothelial cells of blood vessels. The shear-thinning effect of blood under low shear conditions is mainly attributed to the aggregation of red blood cells (RBCs). In this case, RBCs form rouleaux or 3D networks of RBC aggregates. Given that a considerable increase in RBC aggregation is somewhat correlated with CVDs^8^,^9^ RBC aggregation may also play an important role in understanding the etiological cause of CVDs. For these reasons, the accurate measurement of hemorheological properties is essential to understand their role in the occurrence and development of CVDs and microcirculation diseases.

Several techniques have been introduced to effectively measure blood viscosity^10^,^11^,^12^. A H-shaped microfluidic device, composed of two parallel side channels and a bridge channel, can effectively measure blood viscosity under various flow conditions^13^. RBC aggregation can be measured directly (microscopic observation methods)^14^,^15^, or indirectly. The latter includes measurement modalities such as erythrocyte sedimentation rate^16^, light reflection or transmission^17^, ultrasonic^18^,^19^ and electrorheological methods^20^. However, most conventional methods measure hemorheological properties using collected blood.

Hemorheological properties obtained under *in vitro* condition are different from the values measured directly from an animal model because of different experiment conditions^21^. In addition, the aggregability and deformability of RBCs are influenced by external exposure of blood samples^22^. Therefore, the results obtained by *in vitro* measurement need to be validated under *in vivo* conditions. However, it is technically difficult to accurately measure biophysical properties under *in vivo* conditions. To resolve these problems, a rat extracorporeal loop model, which circulates blood through an external network connecting large-scale artery and vein directly, was developed, recently^23^. By inserting an H-shaped microfluidic device into the rat extracorporeal loop, biophysical properties such as viscosity, flow rate, and pressure were measured as a function of elapsed time under *ex vivo* condition^24^,^25^,^26^. However, the flow rate of the blood delivered through the complex network was unspecified. To evaluate the unknown flow rate of blood, additional experimental procedure should be conducted using the blood sample collected from the rat extracorporeal model.

Recently, a high-frequency ultrasound system with speckle image velocimetry (SIV) was demonstrated to measure the velocity fields of blood flows with a reasonable accuracy. It applies a cross-correlation algorithm to ultrasound images of RBCs or RBC aggregates^27^,^28^. In addition, an ultrasound imaging system can capture spatial and temporal distributions of RBC aggregates^19^,^29^. Therefore, the information on the velocity fields of blood flows and the characteristics of RBC aggregation can be simultaneously obtained from the ultrasound imaging system.

A hybrid system composed of an ultrasound imaging system and a microfluidic device connected to a rat extracorporeal model is proposed to monitor temporal variations of hemorheological and hemodynamic properties with better accuracy. This system has several distinctive advantages. First, the biophysical properties, including blood viscosity, mean pressure, blood flow, RBC aggregation, heart rate and temperature, of real blood circulating in the extracorporeal microfluidic loop can be simultaneously measured without noticeable hemorheological changes. Second, this system can be used to sensitively monitor the temporal variations of hemorheological and hemodynamic parameters properties in a short-term period under *ex vivo* conditions such blood dialysis because the proposed system can measure these properties without blood collection from a rat model. The proposed system also might be utilized as a measurement tool for understand the relation between these properties and CVDs. In addition, the hemorheological properties at relatively low and high shear rate conditions can be simultaneously measured using microchannel and vascular phantom with different size. Lastly, the proposed method does not require any calibration procedures because it does not have any flow sensors.

To demonstrate the feasibility and usefulness of the proposed system combined with an ultrasound imaging modality and microfluidic device, the biophysical properties including RBC aggregation, blood flow velocity, and blood viscosity of blood samples are estimated under various *in vitro* conditions. Then, hybrid system is applied to the rat extracorporeal bypass model to monitor temporal variations of biophysical properties.

## Results

### Measurement of biophysical properties using the proposed system

**Figure 1:**
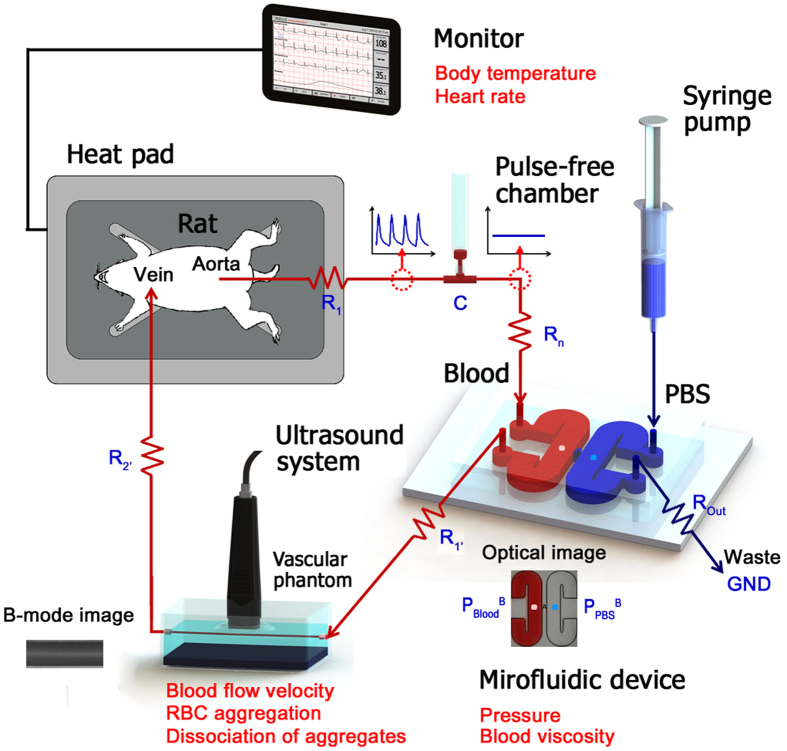
A proposed hybrid system for *ex vivo* monitoring biophysical properties of blood. The system is composed of an ultrasound system and a microfluidic device for monitoring temporal variations of the biophysical properties of blood samples. The extracorporeal bypass loop consists of a pulse-free chamber (air cavity = 0.5 mL), a microfluidic device, and a vascular phantom for ultrasound imaging. Blood is supplied into the fluidic network by connecting the extracorporeal loop to blood vessels of a rat model. The body temperature of a rat model is maintained using a heat pad. To induce the hemodynamic balancing state in the microchannel, PBS solution is delivered as a reference fluid by a syringe pump. A discrete fluidic circuit of the proposed system is composed of fluidic resistances (R_1_^…^R_n_, R_1′_, R_2′_, R_out_), air compliance (C), flow rates (Q_Blood_, Q_PBS_). At the hemodynamic balancing state, the pressures (P_Blood_^B^, R_PBS_^B^) at both ends of the bridge channel are identical.

Figure 1 shows a schematic diagram of the proposed hybrid system to monitor temporal variations of biophysical properties under *ex vivo* conditions. The proposed system consists of a pulse-free apparatus (air cavity = 0.5 mL), a microfluidic device, an ultrasound imaging system, and a heat pad with monitoring system. The pulsatile flow supplied from the aorta is stabilized by passing through the pulse-free chamber. Given that the blood circulating through the extracorporeal network is cooled, a heat pad is employed to maintain the body temperature of the rat model. To measure blood viscosity, a hydrodynamic balancing state is induced in the microfluidic device by adjusting the injection flow rate of a phosphate-buffered saline (PBS) solution with a syringe pump. Ultrasound images of blood flows in the extracorporeal loop are then acquired. The rectal temperature and heart rate are monitored simultaneously to check the biophysical conditions of the rat model.

Hemorheological properties such as the degree and concentration of RBC aggregation, and dissociation of RBC aggregates, and hemodynamic properties, including velocity profile and flow rate are measured by analyzing ultrasound images captured by an ultrasound imaging system. Blood viscosity and hydrodynamic balancing pressure in the microfluidic device are estimated by using the microfluidic device. As depicted in Fig. 1, a discrete fluidic circuit of the proposed system is composed of fluidic resistances, air compliance of the pulse-free apparatus, and flow rates. By adding pressure drops in the fluidic circuit model from hydrodynamic balancing pressure in the microfluidic device, the mean pressure at the abdominal aorta (P_Aorta_) can be estimated using following equation;


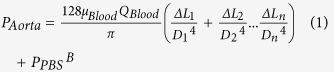


where, L_n_ and D_n_ are the characteristic length and hydraulics diameter of a certain part in the extracorporeal network. P_PBS_^B^ denotes the hydrodynamic balancing pressure of PBS solution. In this numerical prediction, it is assumed that blood viscosity is identical in every parts of a complex extracorporeal network and blood is circulated in the extracorporeal network without bleeding. 

### Estimation of RBC aggregation using ultrasound speckle images

**Figure 2:**
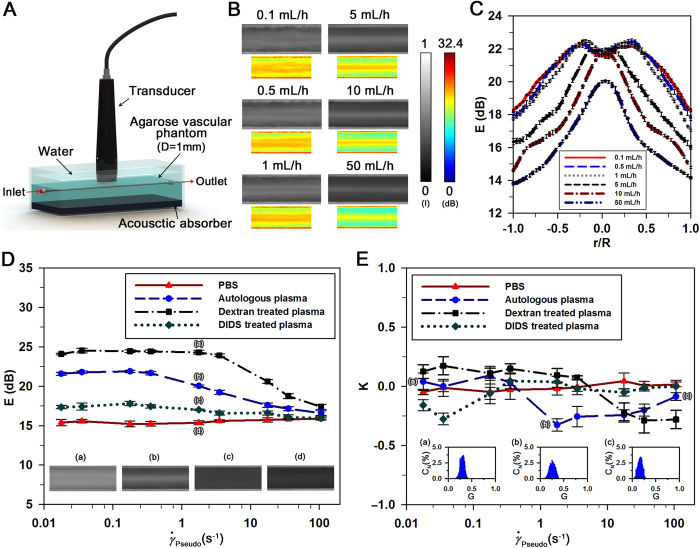
Estimation of RBC aggregation by analyzing ultrasound images of blood flows. (**A**) A schematic of the ultrasound imaging system. Blood is delivered into the vascular phantom with an acoustic absorber to minimize ultrasound artifacts. **(B)** Typical speckle images of blood flows at different flow rates. Speckle images are obtained by averaging a total of 255 ultrasound images. For easy comparison, the corresponding images with false colors representing the logarithm of signal power in decibels are included. Decibel is calculated by dividing blood signals to water signals. **(C)** Radial variations of the mean echogenicity of blood flows shown in Fig. 2B. Error bars indicate the standard deviation (*n* = 255). **(D)** Variations of mean echogenicity (E) in the center of tube (ranged from −0.25 to 0.25 of normalized radius) according to pseudo shear rate (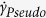) among four different groups. Error bars indicate the standard deviation (*n* = 2). **(a–d)** Ultrasound B-mode images for four different groups at a flow rate of 5 mL/h. **(E)** Variations of kurtosis (K) in the center of tube according to pseudo shear rate. Error bars indicate the standard deviation (*n* = 2). Histograms of echo speckle amplitude data (G) of RBCs in autologous plasma at a flow rate of **(a)** 0.05**, (b)** 5, and **(c)** 300 mL/h.

To monitor temporal variations of RBC aggregation in a vascular phantom, ultrasound system is adopted (Fig. 2A). Given that the ultrasound signal amplitude of blood flows is determined by the size and amount of RBC aggregates, B-mode images show bright speckles at low flow rate conditions as shown in Fig. 2B. Speckle images are depicted in decibels (dB) by evaluating the ratio of the ultrasound signals of blood to PBS signals for clear comparison. Figure 2C shows radial variations of echogenicity obtained by averaging the B-mode images along the flow direction at different flow rates. As the blood flow rate decreases, a normal distribution of echogenicity with high values around the tube center is changed into a blunt shape with higher values. This phenomenon may be attributed to the fact that the decrease of shear force facilitates the formation of RBC aggregation and their propagation from the tube center to the near-wall region.

To quantify the degree of RBC aggregation, mean echogenicities (E) in the tube center (ranged from r/R = −0.5 to 0. 5) of different flow rates are compared (Fig. 2D). A pseudo shear rate (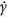
_pseudo_) is adopted to represent shear force in the tube center by using the following equation:


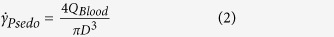


where, D denotes the diameter of a vascular vessel and Q_Blood_ is the blood flow rate. As the pseudo shear rate decreases from 100 to 0.1 s^−1^, the echogenicity drastically increases due to the formation of RBC aggregates. Given that RBC aggregation is more enhanced at a relatively low shear rate of 0.1 s^−1^ compared with no shear condition^30^, the maximum echogenicity of RBCs in autologous plasma is observed around the pseudo shear rate of 0.1 s^−1^. However, for low shear rates ranging from 0.01 to 0.5 s^−1^, the variation of echogenicity is not so significant.

To change hemorheological properties, RBCs suspended in PBS solution, and dextran and 4,4′-diisothiocyanatostilbene-2,2′-disulfonic acid (DIDS) treated plasmas are compared with RBCs in autologous plasma. Considering that the dextran treatment ascertains intercellular interactions between RBCs, the echogenicity of RBCs in the dextran-treated plasma has significantly large values. It is difficult to precisely distinguish the changes in echogenicity for the dextran case under low shear conditions. In contrast, the DIDS treatment effectively inhibits the formation of RBC aggregates, but the echogenicity is slightly increased under low shear rate conditions. Given that RBCs in PBS solution are completely dispersed, the echogenicity is almost similar regardless of shear rate.

Ultrasound blood speckles are composed of many independent scatters including RBCs and RBC aggregates. When backscattered signals have Gaussian distribution, Rayleigh scattering is dominant in the captured ultrasound images according to the central limit theorem^29^. To investigate the concentration of scatters, a statistical analysis is conducted for various speckle images (Fig. 2E). Considering that the kurtosis (K) of Gaussian distribution is zero, the kurtosis for RBCs in PBS solution with similar to zero value can be explained by assumption that completely dispersed RBCs can be considered as Rayleigh scatters. However, this consideration may not always be valid for echogenicity scattered by RBCs or RBC aggregates. As shown in Fig. 2E(a–c), speckle images of many large RBC aggregates exhibits a leptokurtic distribution with a high mean value. As a result, the RBCs in the autologous and dextran-treated plasma have kurtosis values of nearly zeros under very low shear rate conditions. Speckle images of blood with relatively small RBC aggregates have a platykurtic distribution, resulting in negative kurtosis. Specific shear rate condition with a minimum kurtosis is quite different among groups, because chemical treatments with dextran and DIDS change RBC aggregability noticeably. As the shear rate increases beyond the specific shear rate, kurtosis values approach zero because of dispersion of RBC aggregates.

### Velocity field and flow rate of a newtonian fluid

**Figure 3:**
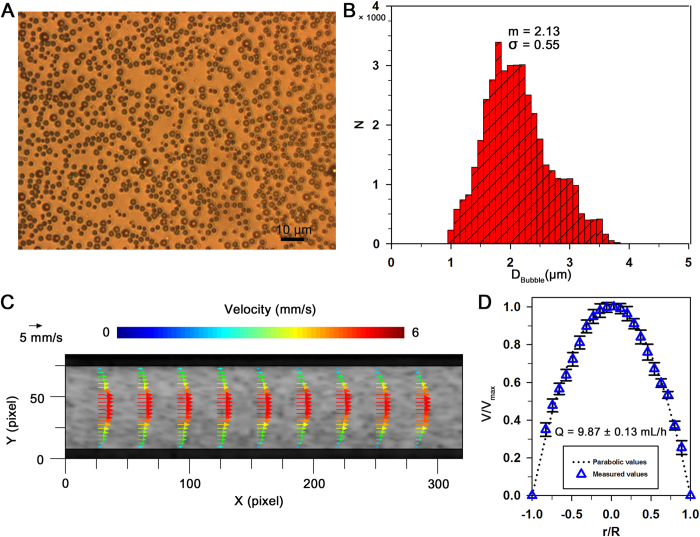
Velocity field measurement of a Newtonian fluid through SIV technique. (**A)** Microbubbles observed by an inverted fluorescence microscope with a 100× objective lens (NA of 1.3). (**B)** Size distribution of microbubbles measured by digital image processing techniques. (**C**) An instantaneous velocity field of PBS solution with microbubbles in the vascular phantom at a flow rate of 10 mL/h. The corresponding ultrasound image is superimposed on the velocity field data. **(D)** Normalized velocity profile (V/V_max_) along the normalized radial direction (r/R). The theoretical parabolic velocity profile is included for comparison. The flow rate of PBS solution is evaluated by Eq. (3).

The performance of the ultrasound imaging system used in this study is validated by applying it to a Newtonian fluid (PBS solution). Given that ultrasound agents have been commonly used for quantitative visualization of opaque flows^31^,^32^,^33^, microbubbles are used as tracer particles to measure velocity field of a PBS flow. As shown in Fig. 3A, the microbubbles used in this study have a hollow structure. Morphological characteristics of the microbubbles are quantified using digital image processing techniques. The mean diameter of the microbubbles is measured as 2.13 μm (Fig. 3B).

PBS solution seeded with microbubbles is delivered by a syringe pump into the vascular phantom at a flow rate of 10 mL/h. Figure 3C shows typical velocity field obtained from ultrasound images of the PBS flow. The velocity profiles are symmetric with respect to the center of the vessel. To compare the measured velocity profiles with the analytical solution, radial velocity profiles are obtained by averaging the entire velocity fields along the flow direction (Fig. 3D). Velocity profile and radial position are normalized by dividing with the maximal velocity and vessel radius, respectively. The flow rate (Q) evaluated by following equation based on a fitting model is compared to injection flow rate of the syringe pump;


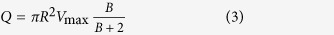


where V_max_ is the maximum velocity at the vessel center, and R represents the vessel radius. A bluntness index (B) describes the flatness of the velocity profile. For instance, the normal velocity profile with a bluntness index of 2 is parabolic. When the bluntness index is larger than 2, the profile has a blunt shape^28^. The flow rate estimated by Eq. (3) is about 9.89 mL/h with a bluntness index of B = 2.01. Therefore, the measured velocity profile is in a good agreement with the analytical solution. This finding implies that the present ultrasound system can be reasonably used to measure velocity field information.

### Blood flows and dissociation of RBC aggregates

**Figure 4:**
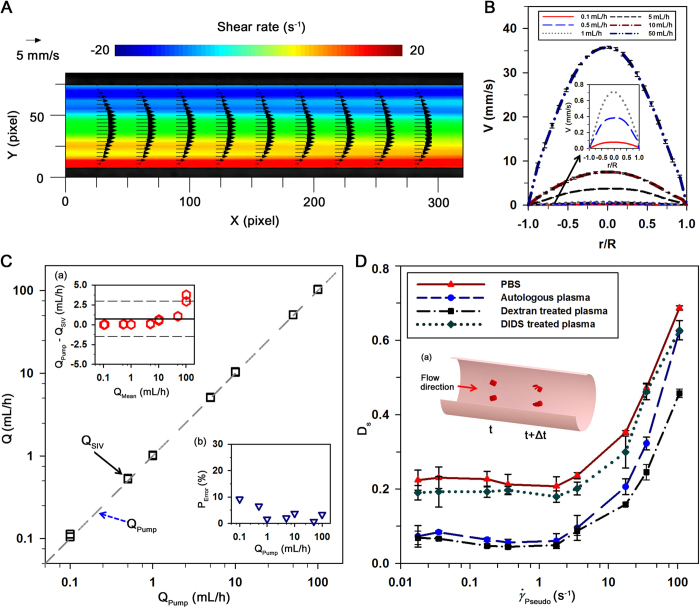
Estimation of blood flow rate and dissociation of RBC aggregates using the SIV technique. **(A)** A typical velocity field and shear rate distribution of a blood flow in the vascular phantom at a flow rate of 10 mL/h. **(B)** Variations of axial velocity profile (V) for RBCs in plasma along the normalized radial direction (r/R) at different flow rates. The magnified velocity profiles at very low flow rates are included. Error bars indicate standard deviation (*n* = 250). **(C)** Comparison of blood flow rates measured through SIV technique (Q_SIV_) and the input flow rate of syringe pump (Q_Pump_) under various flow rate conditions (0.1-300 mL/h). **(a)** The difference between the input flow rate and measured flow rate (Q_Pump _−_ _Q_SIV_) is depicted in the Bland–Altman plot with respect to their average value (Q_Mean_). A bold line and dashed lines denote the mean value and 95% limits of agreement, respectively. **(b)** Variation of percentage error (P_error_ = (Q_Pump _−_ _Q_SIV_)/Q_Pump_) **(D)** Variations of the decorrelation of speckles (D_S_) according to pseudo shear rate (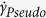) for the four groups. Error bars indicate standard deviation (*n* = 2). **(a)** A schematic representation for the dissociation of RBC aggregates in a blood flow. RBC aggregates aligned with the flow direction at a specific time (t) are dissociated by shear force during the time interval (Δt).

Different from a Newtonian fluid, blood flows are varied according to physiological conditions^26^,^34^. Velocity fields of RBCs in autologous plasma at various flow rates are measured by using the SIV technique^28^,^35^. Figure 4A shows a typical instantaneous velocity field of a blood flow in the vascular phantom at a flow rate of 10 mL/h. Spatial distribution of corresponding shear rate is superimposed on the velocity field. Shear rate has a maximum value at the vascular wall and zero value in the tube center. Figure 4B shows the velocity profiles measured at six flow rates. As expected, the velocity profiles become blunt with decreasing flow rate^26^.

By applying Eq. (3), the flow rate of each blood flow is estimated. Figure 4C compares the estimated flow rates of RBCs in autologous plasma and injection flow rates supplied by the syringe pump. Bland–Altman analysis is adopted for a more detailed comparison of the flow rates estimated by the SIV technique with injection flow rate^36^. In the Bland–Altman plot of Fig. 4C(a), the difference between two techniques is depicted against their average values. A bold line and dashed lines indicate the mean value and ±95% limits of agreement, respectively. Given that the presentation of data inside the 95% limits is utilized to judge the agreement of two methods, the flow rates measured by the SIV technique are in a good agreement with the injection flow rates, except for the high flow rate region. As shown in Fig. 4C(b), percentage errors are also evaluated to compensate for the large variances in high flow rate condition. Unlike the Bland–Altman analysis, the percentage errors at low flow rates have relatively high values although they are less than 10%. This phenomenon results from unstable behaviors of the syringe pump at a low flow rate, and from the significantly small displacement of speckles in ultrasound images.

An additional biophysical parameter about RBC aggregation can also be measured using the present methods. As illustrated in Fig. 4D(a), the spatial distribution of RBC aggregates aligned with the flow direction at a specific time (t) varies due to shear force during the time interval (Δt). Considering that variations in spatial distribution of scatters lead to change of speckle images, the decorrelation of blood speckles (D_S_) based on speckle similarity can be used to estimate the degree of dissociation of RBC aggregates. Figure 4D shows variations of dissociation with respect to the pseudo shear rate for four different groups. As the pseudo shear rate increases beyond a specific value, the decorrelation values for all groups are dramatically raised. Weak intercellular interactions of RBCs in PBS solution and DIDS-treated plasma bring about high decorrelation values over all shear rate conditions. The breakdown of rouleaux into individual cells is restrained in dextran-treated plasma because of enhanced RBC aggregation. Therefore, the decorrelation of speckles for dextran-treated group has the lowest values for all shear rate conditions.

### Blood viscosity

**Figure 5:**
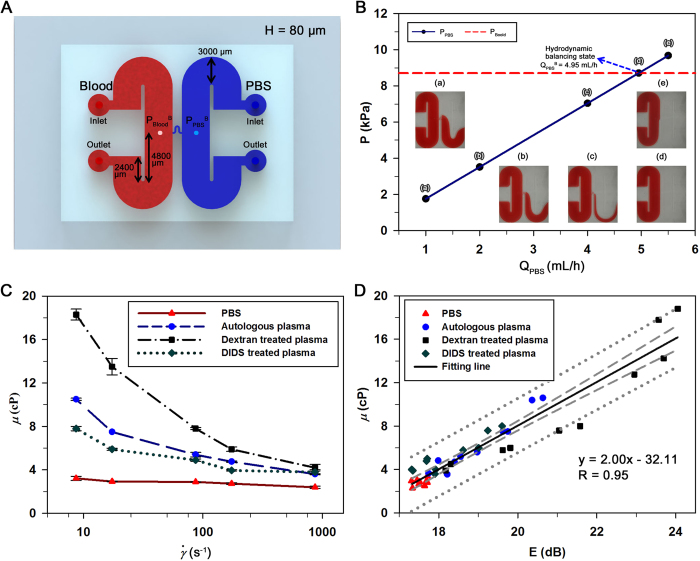
Variations of blood viscosity and its relation to RBC aggregation. (**A**) A microfluidic device with two identical side channels and a connecting bridge channel. The width and height of the channels are 3000 and 80 μm, respectively. Blood sample and PBS solution are delivered into the left and right side channel, respectively. (**B**) Variation of pressure at the right side end of the bridge channel (P_PBS_) according to the flow rate of PBS solution (Q_**PBS**_). Pressure at the left side end of the bridge channel (P_Blood_) is constant, because the blood flow rate is fixed at 1 mL/h. **(a–e)** Microscopic images showing flow conditions in the microfluidic device; the direction of blood flows passing through the bridge channel dependent on the flow rate of PBS solution. At the PBS flow rate of 4.95 mL/h, the pressures at the both ends of the bridge channel reach to the hydrodynamic balancing state. **(C)** Variations of blood viscosity (μ) with respect to shear rate (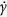) for RBCs suspended in PBS solution, autologous plasma, dextran-treated and DIDS-treated plasmas with a hematocrit of 40%. **(D)** Relationship between the blood viscosity (μ) and echogenicity (E). Solid line represents the linear-curve fitting. R value and the corresponding fitting equation are included. Dashed and dotted lines indicate 95% confidence and prediction intervals, respectively.

Viscosities for all groups (μ) are measured by monitoring the hydrodynamic balancing state in the bridge channel (D_Bridge_) of the microfluidic device (Fig. 5A). When a blood sample and PBS solution are delivered separately into the two inlets of the microfluidic device at the same flow rate of 1_ _mL/h, the pressure of blood sample (P_Blood_) at the left end of the bridge channel is larger than that of PBS solution at the right end on account of different fluidic resistance (Fig. 5B). Thus, the blood sample moves from the left-side to the right-side in the bridge channel, as depicted in Fig. 5B(a). As the PBS flow rate increases, the pressure of PBS solution is also increased and the blood flow passing through the bridge channel decreases. When the pressures of the blood sample and PBS solution at the both ends of the bridge channel reach to a hydrodynamic balancing state, the blood sample does not move to the bridge channel as depicted in Fig. 5B(d). When the pressure of PBS solution becomes higher than that of the balancing state, a reverse flow of PBS solution occurs in the bridge channel, as shown in Fig. 5B(e).

Viscosities of all groups are measured with varying flow rate. Given that blood viscosity is strongly dependent on shear rate, it is depicted with respect to the shear rate (Fig. 5C). The shear rate (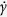) of a blood flow in the microfluidic channel is approximately estimated using the following equation^37^,


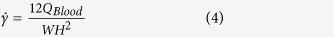


here, W and H denote the width and height of a rectangular channel, which are 3,000 and 80_ _μm, respectively, in this study.

As expected, the sample in the plasma behaves as a non-Newtonian fluid. The viscosities of samples treated with dextran have highest values compared with others over all shear rates because the dextran-treated plasma enhances RBC aggregation. The DIDS-treated plasma degrades the increase of blood viscosity according to the decrease of shear rate by inhibiting the formation of RBC aggregates. On the contrary, the viscosity of the RBCs suspended in PBS solution has a nearly constant value regardless of the shear rate because RBCs are completely dispersed in PBS solution.

### Hemorheological properties under *in vitro* conditions

To investigate the relationship between the blood viscosity and RBC aggregation, viscosities of all samples are plotted against the echogenicity of the samples. Echogenicity averaged over the entire tube is linearly interpolated for matching the shear rates in the microchannel and vascular phantom. As depicted in Fig. 5D, the blood viscosity shows a linear relationship with the echogenicity of blood. A linear curve-fitting method is applied to all data and correlation coefficient value (R) of the fitting line is found to be approximately 0.93. This finding implies that the blood viscosity is highly correlated with RBC aggregation, regardless of blood treatments.

However, the hemorheological properties of blood samples are altered by *in vitro* exposure. To investigate these effects, blood viscosity, echogenicity, and decorrelation of speckles for the whole blood of a rat are measured after *in vitro* exposure of the collected blood samples. Similar to a previous study, the blood viscosity, and degree of RBC aggregation are considerably elevated with lapse of time (Table 1)^22^. This finding may be attributed to the change in the RBC shape from discocyte to echinocyte with the depletion of ATP. Therefore, hemorheological measurement should be carried out under *ex vivo* or *in vivo* conditions in order to more precise measure hemorheological properties.

### Monitoring biophysical properties in the rat extracorporeal loop

**Figure 6:**
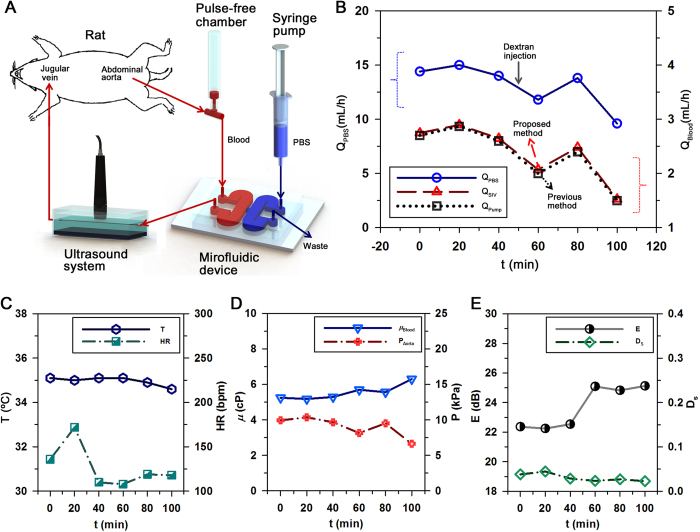
Temporal variations of biophysical properties in the rat extracorporeal model. (**A**) A schematic of the microfluidic system used for monitoring the temporal variations of biophysical properties of blood circulating in the complex fluidic network. The fluidic network is established by connecting the abdominal aorta and jugular vein. (**B**) Temporal variations in flow rate of the PBS solution at the hydrodynamic balancing state (Q_PBS_). The blood flow rates (Q_Blood_) are measured by using the SIV technique (Q_SIV_) and the previous method (Q_Pump_). Exactly blood samples of 2_ _mL are collected at 40 and 100 min to determine the blood flow rates by the previous method. Dextran solution (6%) is injected into the rat model at 50 min to enhance RBC aggregability. (**C**) Temporal variations of the rectal temperature (T) and heart rate (HR). (**D**) Temporal variations of the blood viscosity (μ_Blood_) and mean pressure at the abdominal aorta (P_Aorta_). (**E**) Temporal variations of echogenicity (E) in the center of the tube (ranged from −0.25 to 0.25 of the normalized radius) and the decorrelation of ultrasound speckles (D_S_).

Biophysical properties are repeatedly measured using hybrid technique at intervals of 20 min for a total of 100 min after establishing the extracorporeal rat bypass model (Fig. 6A). For varying hemorheological properties, dextran solution (6%) of 200 mg/kg is injected into the jugular vein of the rats at 50 min. For comparison between proposed technique and a previous method, a blood sample of 2 mL is collected at 40 min and 100 min.

Figure 6B shows temporal variation of flow rate of PBS solution (Q_PBS_) at the hydrodynamic balancing state. By applying the SIV technique to ultrasound speckle images, the flow rate of the bypassing blood flow of the rat model is measured (Q_SIV_). To determine the flow rate at the hydrodynamic balancing state using the previous method, additional measurement procedure is carried out using collected rat blood as introduced in the previous method (Q_Pump_)^25^. Although the flow rates measured by the SIV technique are slightly higher than those estimated by the previous method, the deviations are not so significant. The flow rates of PBS solution and blood samples at the hydrodynamic balancing states are gradually decreased during the experiment.

Figure 6C shows variations in the rectal temperature (T) and heart rate (HR). Although the heat pad is used to maintain the body temperature during the experiment, the body temperature of a rat is slightly decreased from 36 °C to 35 °C due to anesthesia and the extracorporeal circulation of blood. Heart rate is considerably varied from 100 to 180 bpm during the experiment. These values are smaller than that of a normal rat. This variation tendency is slightly correlated with the flow rate of blood (R = 0.56).

By substituting the flow rates of PBS solution and blood at hydrodynamic balancing state into Eq. (7), their blood viscosities (μ_Blood_) are measured at a specific measuring time. Although the injection of dextran and the decrease of flow rate have somewhat influence on the increment in blood viscosity, this variance is not noticeable (Fig. 6D). Pressure has a linear relation with the product of flow rate and viscosity as depicted in Eq. (1), while it is inversely correlated with blood viscosity (R = −0.97). This discrepancy may be caused by large variance in the measured flow rate than blood viscosity. As shown in Fig. 6D, the mean pressure in the abdominal aorta is somewhat decreased at 60 min owing to collection of blood samples.

Hemorheological properties, such as echogenicity (E) and decorrelation of blood speckles (D_S_) can be continuously monitored using the proposed system. As shown in Fig. 6E, the injection of dextran solution at 50 min induces noticeable change in echogenicity, because the enhanced RBC aggregability augments ultrasound signals. However, the effect of dextran treatment on the decorrelation of speckles is not so significant. This results from very low degree of dissociation of RBC aggregates in blood flows at low shear conditions.

## Discussion

RBC aggregation has been regarded as a crucial parameter in determining hemorheological conditions. An ultrasound examination has been widely used to characterize RBC aggregation in blood sample^19^,^29^,^38^. In the present study, RBC aggregations under various hemorheological conditions are estimated by analyzing ultrasound backscattered signals. Mean echogenicity and kurtosis in the center region are used in conducting quantitative analysis on RBC aggregation. The mean echogenicity exhibiting the degree of RBC aggregation is largely varied according to hemodynamic and hemorheological conditions (Fig. 2D). As shown in Fig. 2E, the kurtosis becomes zero when Rayleigh scattering is dominant in ultrasound signals. It is useful to figure out the concentration of RBC aggregates. However, the high degree of RBC aggregation under very low shear rate conditions also give rises to zero kurtosis. This implies that kurtosis is not a suitable parameter for monitoring the hemorheological variations in the rat extracorporeal model.

To measure the hemodynamic and hemorheological parameters of blood circulating in the rat extracorporeal loop without any collection of blood samples, the blood flow rate has to be accurately measured. The ultrasound system used for the flow measurement is validated using a Newtonian fluid and blood samples. Velocity profiles and flow rates of the PBS solution measured through the SIV technique show good consistency with the analytical solution and injection flow rate, respectively (Fig. 3). In addition, the blood flows measured by the SIV technique reasonably coincide with the flow rates supplied by the syringe pump (Fig. 4C). Since this ultrasound system can measures the hemodynamic and hemorheological information of blood flows simultaneously, an additional parameter of the decorrelation of speckles can be obtained. Rouleaux are separated into either individual RBCs or small RBC aggregates when they are subjected to a shear force^18^,^39^. In the present study, the dissociation of RBC aggregates is estimated by analyzing the decorrelation of speckles. The low degree of decorrelation of speckles at a specific shear rate condition indicates a strong intercellular interaction of RBCs.

By monitoring the hydrodynamic balancing state in the microfluidic device, the temporal variations of blood viscosity are measured with high accuracy (Fig. 5). Since the blood viscosity and RBC aggregation of the same blood samples are measured by microfluidic device and ultrasound imaging system, their relation can be investigated directly (Fig. 5D). As a result, the blood viscosity is highly correlated with the mean echogenicity. This high correlation between blood viscosity and mean echogenicity supports that the increase of RBC aggregation augments blood viscosity, regardless of intercellular interaction of RBC and applied shear force.

After validating measurement accuracy of the combined techniques for RBC aggregation, blood flow rates, and blood viscosity under *in vitro* conditions, temporal variations of biophysical parameters are monitored under *ex vivo* condition (Fig. 6). To compare the measurement performance of the proposed method with that of previous method, blood sample is collected from the rat extracorporeal model during the experiment because the previous method found the flow rate of blood at the hydrodynamic balancing state using collected blood sample. In light of hemodynamic properties, the heart rate, blood flow rate and mean pressure at the abdominal aorta are considerably varied during the experiment. This kind of considerable variations may be attributed to the bleeding at coupling joints, anesthetization, environmental stress, and external collection of blood samples^38^.

Especially, the external collection of blood samples makes it difficult to accurately detect the temporal variations of hemorheological properties^25^. When hemorheological conditions are consistently varied in the rat extracorporeal model due to chemical treatments, previous methods should collect blood samples from the rat model at measurement instances. However, continuous collection of blood samples modifies the hemorheological properties of the circulating blood and ultimately induces biophysical damage to the rat model. In addition, the hemorheological properties are directly influenced by the *in vitro* exposure of blood samples, as shown in Table 1^22^. Considering that the method proposed in this study could consistently measure hemorheological properties without any blood collection, it might be useful to investigate the effects of chemical treatments on the hemodynamic and hemorheological characteristics in short-term.

In addition, the proposed system has a decisive advantage of providing information about the hemorheological properties of a rat model. Specifically, the information on blood viscosity at a high shear condition can be obtained because blood flows are exposed to a noticeably high shear force that is caused by small channel size of the microfluidic device. In contrast, the shear rate induced in the vascular phantom for ultrasound imaging is relatively small. This shear rate can be easily controlled by changing the region of interest for the analysis of RBC aggregation. Taking into account of high correlation between blood viscosity and RBC aggregation (Fig. 5D), the hemorheological properties of blood flows can be simultaneously measured at different shear conditions. This measurement under various shear rate conditions can easily and accurately distinguish the variations of hemorheological properties. For example, the injection of dextran solution does not induce a noticeable change in blood viscosity. However, mean echogenicity is considerably increased by dextran treatment. Thus, the elevated RBC aggregation can be easily detected by monitoring ultrasound signals.

The feasibility and measurement accuracy of the proposed system for monitoring temporal variations of the various biophysical properties under *ex vivo* condition are demonstrated. However, the cannulation of large-size abdominal aorta in a rat model to supply blood to the extracorporeal loop gives rise to physiological limitation in long-term monitoring of various biophysical properties. To investigate the effects of drugs or chemicals for long-term treatment on hemorheological properties using present experimental system, a series of the same experiment using rat samples having different drug administration times should be carried out or this system has to be improved to apply to large-size animal models such as fig or dog. Because large-size animals circulate a large amount of blood with high blood pressure, it is possible to supply test blood to the extracorporeal loop by cannulation between the small vein and artery. The carefully handling large animals helps to monitor the temporal variations of hemodynamic and hemorheological properties for relatively long period of time. Besides a cannulation, measurement modalities of this hybrid system should be integrated into one device for clinical applications. From these improvements, this system would be useful for better understanding of the relationship between CVDs and biophysical parameters, and it can be utilized as a complementary diagnostic modality to monitor the variations in hemorheological and hemodynamic parameters under *ex vivo* conditions such as blood dialysis.

## Methods

### Blood sample preparation

In this study, male Sprague-Dawley (SD) rats with body weight in the range of 398–416 g are tested. All blood samples, collected through an abdominal aortic puncture under intramuscular ketamine (100 mg/kg) and xylazine (10 mg/kg) anesthesia, are anticoagulated with ethylenediaminetetraacetic acid (EDTA) dipotassium salt (1.5 mg of EDTA per 1 mL of blood). The blood samples are divided into four groups: RBCs suspended in PBS (pH 7.4, Bio Solution, Korea), autologous plasma, dextran-treated plasma and DIDS-treated plasma. Each group has two samples. For the DIDS-treated sample, blood is taken at 30 min after injecting 50 mg/kg of DIDS to jugular vein^19^. The collected blood samples are separated into RBCs and plasma through centrifugation. Subsequently, the buffy layer is removed. Dextran 500 (molecular mass 450–550 kDa; Sigma) in PBS (6%) is mixed with plasma to archive a plasma-dextran concentration of 0.6% in the dextran-treated blood sample. The hematocrit of all blood samples is precisely adjusted to be 40% by carefully mixing RBCs with four different solutions (PBS solution, autologous plasma, plasma-dextran and plasma-DIDS mixtures). All procedures performed on the animals are approved by the Animal Care and Ethics Committee of POSTECH and the methods are carried out in accordance with the approved guidelines.

### Fabrication of the microfluidic device

A rectangular master replica molder (height = 80 μm) is fabricated using MEMS technologies based on soft lithography process and deep reactive-ion etching. The microfluidic device has two identical side channels (width = 3000 μm, length = 14.4 mm) connected by a bridge channel (width = 100 μm, length = 2.4 mm) as shown in Fig. 5A. After pouring polydimethylsiloxane (PDMS) (Sylgard 184, Dow Corning, USA) is poured on the silicon molder, it is cured at 80 °C for 3 h. Thereafter, a PDMS block is peeled off from the silicon molder. Two side channels have their respective inlet and outlet made of a 1 mm diameter puncher. After oxygen-plasma treatment (CUTE, Femto Science, Korea), the microfluidic device is finally prepared by bonding the PDMS block with a glass substrate. Prior to the experiments, channels of the microfluidic device are incubated with 2% bovine serum albumin (Sigma, MO) for 10 min at room temperature to prevent adhesion of cells.

### Experimental setup

Figure 2A shows the experimental setup for ultrasound imaging. A total 255 of ultrasound speckle images are obtained with a 35-MHz mechanical sector-scan probe (Capistrano Labs., San Clemente, CA, USA) at a frame rate of 50 fps with a sector angle of 20°. To minimize the acoustic discrepancy caused by the tube wall, a vascular phantom with an inner diameter of 1000 μm is adopted to acquire ultrasound images. The details of the vascular phantom are described in our previous study^28^. The measurement section over the agarose vascular phantom is covered with water to facilitate acoustic transmission. The center of the lumen is positioned at the focal length of the transducer (11.8 mm). The transducer chamber, filled with degassed water, is covered with a thin membrane.

Blood sample is supplied into the vascular phantom and the left-side channel of the microfluidic device through syringe pump (neMESYS, Centoni Gmbh, Germany) with a 5 mL plastic syringe (BD). PBS solution is delivered into the right-side channel of the microfluidic device as a reference fluid through the syringe pump. The flow rates of PBS solution are adjusted according to the designed flow conditions for each blood sample. To monitor the flow conditions in the microfluidic device, the microfluidic device is mounted on a stereo microscope (Stemi 2000-C, Zeiss, Germany) equipped with a digital camera (D700, Nikon, Japan). Optical images of the microfluidic device are captured with a magnification of 0.8×. All experiments are conducted at room temperature (25 °C).

### Speckle image velocimetry (SIV)

Velocity fields of the PBS and blood flows are measured using a SIV technique based on a cross-correlation algorithm. Before applying the SIV technique to speckle images, the ultrasound images captured around the blood vessel are cropped into the images of 320 × 84 pixels in size. To enhance the measurement performance of the SIV technique, the captured images are processed by applying digital image-processing techniques and a binary mask is applied to the near-wall region. The detailed procedure and the image-processing techniques used in the present study are well described in our previous studies^26^,^28^,^35^. The size of the interrogation window along the radial direction is fixed to six pixels (0.11 mm in physical dimension) with 50% overlapping. Since the stream wise velocity is largely varied according flow rates, interrogation windows of various sizes are adopted along the flow direction. The obtained velocity vectors are filtered using a 3 × 3 Median kernel to minimize errors.

### Ultrasound agent

To demonstrate the measurement accuracy of the ultrasound system, a flow of PBS solution seeded with ultrasound agent of Sonazoid (GE Healthcare, Oslo, Norway) is measured through SIV technique. A Sonazoid of 0.5% concentration is circulated using a syringe pump. For size measurement of microbubbles, optical images of microbubbles observed by an inverted optical microscope (Zeiss, Germany) with 100×objective lens are converted into binary images with an optimal thresholding value determined through Otsu’s algorithm^40^. A relative diameter is evaluated using the area of microbubbles in binary images. Figure 3B shows the size distribution of microbubbles with mean and standard deviations of 2.13 and 0.55 mm, respectively.

### Data analysis for ultrasound blood speckles

Statistical analysis of ultrasound signals has been used to investigate RBC aggregation^19^,^29^. The coefficient of Kurtosis (K) which represents the peakedness of the probability distribution of speckle image, is calculated using the following equation:


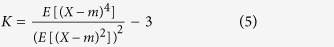


where E is the expectation operator, X is the uncompressed envelop amplitude, and *m* denotes the mean value. A leptokurtic distribution which has an acute peak around the mean value provides a positive Kurtosis. In contrast, a platykurtic distribution induces a negative Kurtosis.

A decorrelation of blood speckles (*D*_S_) based on the speckle similarity can be used to estimate the dissociation of RBC aggregates during a given time interval (Δt)^35^. At first, tile 1 is selected at the center of a speckle image. To compensate the decorrelation caused by the movement of speckle patterns, the tile 2 in the subsequent image is shifted as much as the displacement of speckles estimated through the SIV technique. The decorrelation of blood speckles is calculated by subtracting structural similarity index^41^ between the two tiles from 1 as follows;


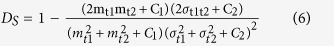


where *m*_t1_, *m*_t2_ and σ_t1_, σ_t2_ denote the mean intensity and standard deviation of tiles 1 and 2, respectively. σ_t1t2_ denotes the covariance of tiles 1 and 2. C_1_ and C_2_ are two small positive constants and they are normally set to be 0.01 and 0.03, respectively. The size of the two tiles used in this analysis is 32 × 32 pixels (0.6 × 0.6 mm).

### Viscosity estimation

Given that the pressures at two identical side channels are identical at hydrodynamic balancing state, the viscosity for blood sample (μ_Blood_) is simply estimated based on the following analytical formula^13^,^24^,


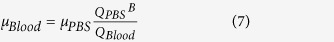


Here, Q_Blood_, Q_PBS_^B^ denote the flow rate of blood sample and PBS solution at the hydrodynamic balancing condition, respectively. Viscosity of the PBS solution (*μ*_PBS_) is approximately 1.00 ± 0.05 cP^24^.

### Variation of hemorheological properties after *in vitro* exposure

Whole blood samples of two rats are extracted through abdominal aortic puncture. Blood viscosity and RBC aggregation are measured at a shear rate of 86.8 and 1.8 s^−1^, respectively. Mean value of hematocrit for the blood samples is 48.0 ± 1.5.

### Preparation of a rat extracorporeal loop model

A male SD rat (16 weeks old, body weight of 427.8 g) is anesthetized with intramuscular injection of ketamine (100 mg/kg) and xylazine (10 mg/kg). A PE-50 (ID = 0.58 mm, polyethylene tube) tube at one end of the bypass loop is cannulated into the right jugular vein. Although there are lots of academic controversies over the use of heparin as an anti-coagulant in the measurement of hemorheological properties, heparin has been commonly and essentially injected into *ex vivo* animal models to prevent blood coagulation inside vascular conduits. The exact amount of heparin (1500 IU/mL/kg) is precisely injected into the right jugular vein. After 10 min of heparin injection, a 22G catheter is inserted into the abdominal aorta. Prior to the connection of extracorporeal conduits to the rat model, the conduits are incubated with 2% bovine serum albumin (BSA, Sigma, MO) for 1 h at room temperature to block platelet deposition and fibrin adhesion. A silicon tube (ID = 0.8 mm) at the other end of the loop is connected with the catheter. As shown in Fig. 6A, pulsed-free microfluidic device with an air cavity of 0.5 mL is connected between the aorta and the inlet of the microfluidic device to supply stabilized blood sample. A vascular phantom is connected with the outlet of the microfluidic device to estimate the hemodynamic characteristics of blood flow and RBC aggregation through the ultrasound system. The blood passing through the extracorporeal conduits is returned to the jugular vein of the rat model. For this, the outlet of the vascular phantom is connected with another PE-50 tube. Since blood cooled by extracorporeal circulation can cause hypothermia, biophysical properties including rectal temperature, heart rate, and electrocardiogram are monitored using a physiological monitoring unit (MouseMonitor, Indus Instruments, USA). Based on these information, the temperature of animal model is maintained by using a heat pad to inhibit hypothermia that can be possibly caused during *ex vivo* measurement. However, the environmental room temperature which is different from body temperature can induce overestimation of blood viscosity and underestimation of RBC aggregation, compared to the hemorheological properties measured under *in vivo* conditions. Preliminary experimental results show that blood viscosity at a shear rate of 8.68 s^−1^ is reduced approximately 10.4%, while echogenicity at a shear rate of 0.28 s^−1^ is increased approximately 1.6%, as the environmental temperature is increased from 25 °C to 37 °C. All experimental procedures are approved by the Animal Care and Ethics Committee of POSTECH and the methods are carried out in accordance with the approved guidelines.

## Additional Information

**How to cite this article**: Yeom, E. *et al.* Hybrid System for *Ex Vivo* Hemorheological and Hemodynamic Analysis: A Feasibility Study. *Sci. Rep.* **5**, 11064; doi: 10.1038/srep11064 (2015).

## Figures and Tables

**Table 1:** Variation of hemorheological properties in rat blood with the lapse of time after *in vitro* exposure.

	0 h	0. 5 h	1 h	2 h	3 h
μ (cP)	6.4_ _±_ _0.2	7.4_ _±_ _0.2	8.2_ _±_ _0.1	11.0_ _±_ _0.3	11.8_ _±_ _0.1
E (dB)	20.09_ _±_ _0.15	20.28_ _±_ _0.13	20.42_ _±_ _0.13	20.94_ _±_ _0.23	21.14_ _±_ _0.11
D_s_	0.0644_ _±_ _0.006	0.0551_ _±_ _0.005	0.0503_ _±_ _0.003	0.0481_ _±_ _0.005	0.04423_ _±_ _0.004

μ, E and D_s_ indicate the blood viscosity, the mean echogenicity and the deccorealtion of speckles. Each value represents mean ± standerd deviation.
